# Cultivation-Dependant Assessment, Diversity, and Ecology of Haloalkaliphilic Bacteria in Arid Saline Systems of Southern Tunisia

**DOI:** 10.1155/2013/648141

**Published:** 2013-11-10

**Authors:** Darine El Hidri, Amel Guesmi, Afef Najjari, Hanen Cherif, Besma Ettoumi, Chadlia Hamdi, Abdellatif Boudabous, Ameur Cherif

**Affiliations:** ^1^Laboratory of Microorganisms and Active Biomolecules, Faculty of Sciences of Tunis, University of Tunis El Manar, 2092 Tunis, Tunisia; ^2^LR Biotechnology and Bio-Geo Resources Valorization, Higher Institute for Biotechnology, Biotechpole Sidi Thabet, University of Manouba, 2020 Ariana, Tunisia

## Abstract

Haloalkaliphiles are polyextremophiles adapted to grow at high salt concentrations and alkaline pH values. In this work, we isolated 122 haloalkaliphilic bacteria upon enrichments of 23 samples from 5 distinct saline systems of southern Tunisia, growing optimally in media with 10% salt and at pH 10. The collection was classified into 44 groups based on the amplification of the 16S–23S rRNA internal transcribed spacers (ITS-PCR). Phylogenetic analysis and sequencing of the 16S rRNA genes allowed the identification of 13 genera and 20 distinct species. Three gram-positive isolates showing between 95 and 96% of 16S rRNA sequence homology with Bacillus saliphilus could represent new species or genus. Beside the difference in bacterial diversity between the studied sites, several species ecological niches correlations were demonstrated such as *Oceanobacillus* in salt crust, *Nesterenkonia *in sand, and *Salinicoccus* in the rhizosphere of the desert plant *Salicornia*. The collection was further evaluated for the production of extracellular enzymes. Activity tests showed that gram-positive bacteria were mostly active, particularly for protease, lipase, DNase, and amylase production. Our overall results demonstrate the huge phenotypic and phylogenetic diversity of haloalkaliphiles in saline systems of southern Tunisia which represent a valuable source of new lineages and metabolites.

## 1. Introduction

Extreme environments are distributed on Earth which were thought to prevent the existence of life. These habitats are characterized by extreme conditions including physical (temperature and pressure) and chemical parameters (salinity and pH) [1]. Major categories of extremophiles include halophiles, thermophiles, acidophiles, alkaliphiles, and haloalkaliphiles. The microflora of high salinity ecosystems has attracted a great deal of attention from researchers in this last decade, especially haloalkaliphiles bacteria. In 1982, the term haloalkaliphile was used for the first time to describe bacteria that are both halophilic and alkaliphilic [2]. This group of bacteria is able to grow optimally or very well at pH values at or above 10 along with high salinity (up to 25% (w/v) NaCl) [3]. To adapt in such conditions, haloalkaliphilic microorganisms have developed various physiological strategies to maintain their cell structure and function [4, 5]. These bacteria have largely been identified and studied from the hypersaline environments, soda lakes, solar saltern, salt brines, carbonate springs, and Dead Sea [6]. Their existence clearly indicated the widespread distribution of such organisms in natural saline environments [4, 7].

During the past years special attention has been focused on the distribution of haloalkaliphilic bacteria diversity in different hypersaline and hyperalkaline environments [8]. Culture based methods are usually used to investigate biochemical and genetic diversity by selecting a particular population of microorganisms [9]. Molecular microbial surveys based on 16S rRNA gene have been adopted to study the phylogenetic diversity in different extreme environments [10–14]. Generally, saline systems are dominated by representatives of the domain Bacteria [15–21]. They possess special adaptation mechanisms to survive, grow, and thrive under high salinity and alkaline pH. This dual extremity of halophile and alkaliphile makes these microorganisms very interesting from the fundamental and biotechnological research sides [22]. 

The interest in haloalkaliphilic microorganisms is due not only to the necessity for understanding the mechanisms of adaptation to multiple stresses and detecting their diversity, but also to their possible application in biotechnology. Research efforts focused on the discovery of industrial enzymes capable of performing their function under harsh conditions have greatly increased over the past decade [7, 22, 23]_._ These enzymes include proteases, lipases, amylases, and DNase, viewed as important candidates for various industries such as food, detergent, chemical, pharmaceutical, paper, and pulp or waste treatment [4]. Southern Tunisia features numerous ecosystems including coastal and inland salt lakes, respectively, named Sabkha or Sabkhet, and Chotts [24]. These environments are characterized by unstable climatic conditions, due to the periodic flooding by the subsurface ground water associated with high salt conditions during dry phases, making them fascinating ecosystems to study the diversity and the ecological adaptations of microorganisms thriving in saline systems. 

To our knowledge, no studies have been carried out in order to describe the diversity of haloalkaliphilic bacteria from North African arid and hypersaline systems. The present work aimed to evaluate the diversity of haloalkaliphilic strains isolated from the inland Chotts and the coastal Sebkha hypersaline systems in Tunisian Sahara, based on different phylogenetic markers and biochemical patterns. 

## 2. Materials and Methods

### 2.1. Sample Collections

**Figure 1:**
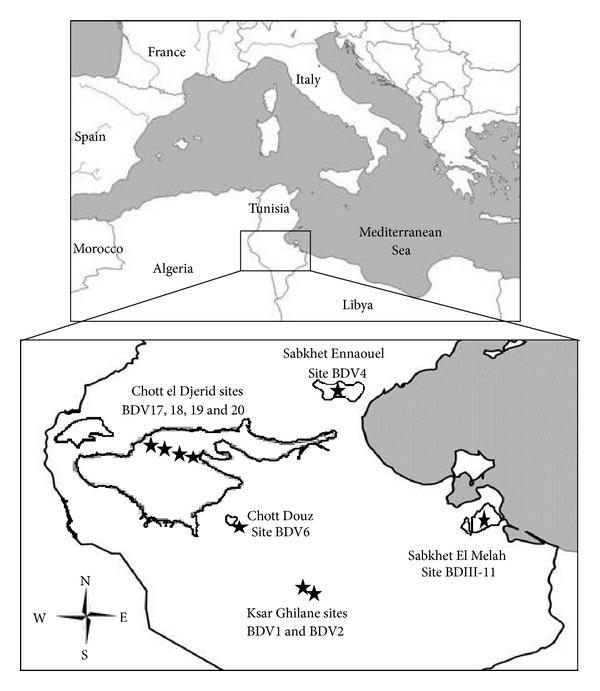
Location of the sampled sites: BDV1 and BDV2 (Oasis Ksar Ghilane), BDV4 (Sabkhet Ennaouel), BDV6 (Chott el Douz), BDV17, BDV18, BDV19, BDV20 (Chott el Djerid), and BDIII-11 (Sabkhet El Melah).

All enrichments and strains described here were isolated from twenty-three samples collected from arid saline systems in southern Tunisia during February 2008 and 2010: salt crust, hypersaline water, thermomineral water, sand, sediment (with or without salt), bulk soil, algal biofilm, and the rhizosphere of the desert plant *Salicornia* when present. The sampling sites include three continental ephemeral salt lakes: Chott el Djerid (9 samples from 4 sites: BDV17, N 33°59′558′′, E 08°39′212′′; BDV18, N 33°58′736′′, E 08°20′632′′; BDV19, N 33°57′252′′, E 08°24′507′′; BDV20, N 33°57′252′′, E 08°24′508′′), Chott el Douz (3 samples from site BDV6: N 33°28′204′′, E 08°56′733′′), and Sabkhet Ennaouel (2 samples from BDV4: N 34°26′951′′, E 09°54′102′′); one coastal salt lake, Sabkhet El Melah (4 samples from BDIII-11: N 33°25′119′′, E 11°00′523′′), and one nonsaline system; Ksar Ghilane Oasis (5 samples from 2 sites: BDV1, N 32°59′012′′, E 09°38′072′′; BDV2, N 32°59′293′′, E 09°38′374′′) (Figure 1). Samples were collected into sterile flasks and kept aseptically at 4°C until analyzed.

### 2.2. Enrichment and Isolation of Haloalkaliphilic Bacteria

Enrichment was performed on Soap lake Basal Medium (SLBM) [25], an enrichment medium for moderately haloalkaliphilic bacteria, containing (L^−1^): CaSO_4_ 4 mg; FeSO_4_ 1 mg; NaCl 10 g; SiO_2_ 5 mg; MgCl_2_ 4 mg; MnSO_4_ 4 mg; NH_4_O_3_ 50 mg; Na_2_SO_4_ 13 g; KH_2_PO_4_ 3 g; K_2_HPO_4_ 3 g; Na_2_CO_3_ 1 g, and 1 mL trace element stock solution consisting of (L^−1^) sodium nitriloacetate 1.5 g; MgSO_4_·7H_2_O 3 g; MnSO_4_·7H_2_O 0.5 g; NaCl 1 g; FeSO_4_·7H_2_O 0.1 g; CaCl_2_·2H_2_O 0.1 g; CoCl_2_·6H_2_O 0.1 g; ZnCl_2_ 0.13 g; CuSO_4_·5H_2_O 0.01 g; AlK (SO_4_)_2_·12H_2_O 0.01 g; H_3_BO_3_ 0.01 g; Na_2_MoO_4_·2H_2_O 0.025 g; NiCl_2_·6H_2_O 0.024 g, and Na_2_WO_4_·2H_2_O 0.025 g. The final pH of the medium was adjusted to 10 by adding 5 M NaOH before autoclaving. One g or 1 mL of each sample was added to 20 mL of SLBM and incubated in a shaking incubator (200 rpm) at 30°C for 5 days. Serial dilutions of the enriched cells were plated on solid SLBM [25]. Plates were incubated at 30°C for 5 days. Colonies growing on the plates were selected based on morphological features, considering pigmentation and size. Each isolate was subjected to successive streak plating until a pure colony was obtained. The isolates were stored in glycerol stocks (25% v/v) at −80°C. 

### 2.3. DNA Extraction and PCR Conditions

Genomic DNA of bacteria was extracted by sodium dodecyl sulfate-proteinase K treatment [26]. The 16S rRNA gene from pure cultures was amplified as a 1.5 kb DNA fragment by PCR using the universal primers S-D-Bact-0008-a-S-20 (5′-CTA CGG CTA CCT TGT TAC GA-3′) and S-D-Bact-1495-a-S-20 (5′-AGA GTT TGA TCC TGG CTC AG-3′) [26]. 16S–23S rRNA ITS were amplified using the universal primers S-D-Bact-1494-a-20 (5′-GTC GTA ACA AGG TAG CCG TA-3′) and L-D-Bact-0035-a-15 (5′-CAA GGC ATC CAC CGT-3′) [27]. PCR amplification was carried out according to the procedure described previously [26]. The presence of specific PCR products was verified by electrophoresis on 1.5% and 2% (w/v) agarose gels for 16S rRNA and ITS amplicons, respectively.

### 2.4. Sequencing and Phylogenetic Analysis of 16S rRNA Sequences

The 16S rRNA gene sequencing has been carried with an automated capillary ABI Biosystem 3130. The obtained sequences were identified by comparison with those available at the National Centre for Biotechnology Information (NCBI) database (http://www.ncbi.nlm.nih.gov) using the BLAST program [28]. The sequences were aligned using Clustal W version 1.8 [29]. Evolutionary distances were computed using Jukes and Cantor method [30]. Phylogenetic dendrograms were constructed by the neighbor-joining method and trees topology was evaluated by performing bootstrap analysis of 1000 data sets using MEGA 4.1 (Molecular Evolutionary Genetics Analysis) [31]. The sequences reported in this study have been submitted to NCBI GenBank and the accession numbers are listed in Table 1. 

### 2.5. Morphological and Physiological Characterization of Isolates

Gram staining of all isolates was performed according to the method of Murray and colleagues [32]. Growth of strains at different pH values was determined in solid SLBM, in which the pH was adjusted to 7.0, 10, and 11. The ability of strains to grow at different range of salinity at pH 10 and pH 7 was performed in solid SLBM plates supplemented with 0, 5, 10, 15, 20, and 25% NaCl (w/v). Growth behaviors were observed after 5 days of incubation at 30°C. 

### 2.6. Screening of Strains for Extracellular Hydrolytic Activities

A qualitative screening was performed to detect the ability of the isolated bacteria to produce extracellular enzymes responsible for hydrolytic activities. The tests were performed on different solid media containing 10% NaCl at pH 10. For alkaline protease detection, SLBM agar medium supplemented with 1% (w/v) skim milk was used as described previously [4, 33]. A clear zone around the colony after 5 days of incubation was taken as evidence of proteolytic activity. Amylase activity was performed according to the method described by Amoozegar and colleagues [34]. The presence of amylolytic activity on plates was determined qualitatively using SLBM agar medium supplemented with 0.5% (w/v) soluble starch. After incubation at 30°C for 5 days, the plates were flooded with 0.3% I_2_-0.6% KI solution. A clear zone around the growth indicated the hydrolysis of starch [35]. DNase activity of the strains was determined using DNase test agar medium. After incubation at 30°C for 5 days, the plates were flooded with toluidine blue (0.1%) (w/v). A pink halo around the colonies showed the secretion of DNase [36]. Lipase screening was achieved based on the method of Gutiérrez and González [37] using Tween 20 as a substrate. The presence of lipase activity was demonstrated by the formation of white halo due to the formation of precipitates of calcium laurate around the growth after 5 days of incubation at 30°C.

## 3. Results and Discussion

### 3.1. Isolation and Characterization of Haloalkaliphilic Bacteria

The diversity of cultivable haloalkaliphilic bacteria was evaluated using culture enrichment followed by isolation on haloalkaliphile medium. A total of 23 samples collected from 4 distinct saline stations (Sabkhas and Chotts) and one desert station were processed. The morphological characteristics of the isolates showed a wide variability including size, color, and margin, with 76.15% of them being Gram-positive. The versatility to grow in different range of NaCl concentrations and pH values is reported in Table 2. On the basis of their salt tolerance, the collection could be classified into three groups: extremely halotolerant (growing at NaCl concentration ranging from 0 to 25%), moderate halotolerant (growing between 0 and 10% NaCl), and strict halophilic bacteria (i.e., that cannot be cultured without salt). Similarly, depending on their tolerance to pH, strains can be divided into two groups: facultative alkaliphile which represent the dominant fraction of the collection (81.15%) and obligate alkaliphile bacteria (18.85%). 

Extremely halotolerant bacteria, in which their salt tolerance ranged between 0 and 15, 20, or 25% (w/v) represented the major part of this collection (71.3%). In similar studies, strains isolated from alkaline Lonar lake in India [7] and from mineral pool in Campania (southern Italy) [38] were shown to be extremely haloalkalitolerant, tolerating high concentrations of NaCl up to 25% and different pH values (7–10).

Combining the salt and pH requirements and their effects on the growth, the group of bacteria that could be considered as obligate haloalkaliphiles represent 24.5% (*n* = 30) of the collection. They were mainly isolated from the extreme saline systems of Chott el Djerid, Sabkhet El Melah, and Chott el Douz (Tables 1 and 2). The ability of haloalkaliphilic strains to grow at a wide range of salinities and pH could be assigned to their adaptation to the changing levels of salinity and by evolving typical strategies to cope with salt stress: osmoregulation and modification in cell morphology and structure [39, 40]. It is interesting to note that a subcollection (23 isolates) of the obligate haloalkaliphiles showed variability in their salt tolerance with different pH values. At alkaline pH (10-11), they were able to cope with the absence of salt, but at neutral pH 7, they require an amount of NaCl higher than 1%. They were thus considered as strict halophilic bacteria at neutral pH. Only 7 isolates (5.73%) from Sabkhet El Melah and Chott el Douz were shown to be strict halophiles at all pH values (Tables 1 and 2). The exact relation between the salt requirement and tolerance and the pH homeostasis in the cell, raises several questions and represents an interesting issue to be studied [7]. Studies on aerobic alkaliphilic bacteria thriving in alkaline Lonar Lake in India showed that obligate haloalkaliphles related to the genus *Alkalibacillus* could be isolated only in specific medium containing 2% NaCl and at pH 10 [7]. 

A fraction of 4.1% of our collection was classified as moderate haloalkaliphiles (0–10% of NaCl growth range), a proportion similarly isolated from other different saline and alkaline environments [41–47]. Occurrence of haloalkalitolerant, obligate, and moderate haloalkaliphiles bacteria, in different sampling locations, highlighted the diversity and the widespread distribution of these microorganisms in arid-saline systems of southern Tunisia. This versatility of growth characteristics could be explained by their ability of osmoregulation, in relation with alkaline pH, through which they maintain an internal osmotic potential that equals their external environment [48].

### 3.2. Bacterial Collection Dereplication and Identification

**Figure 2:**
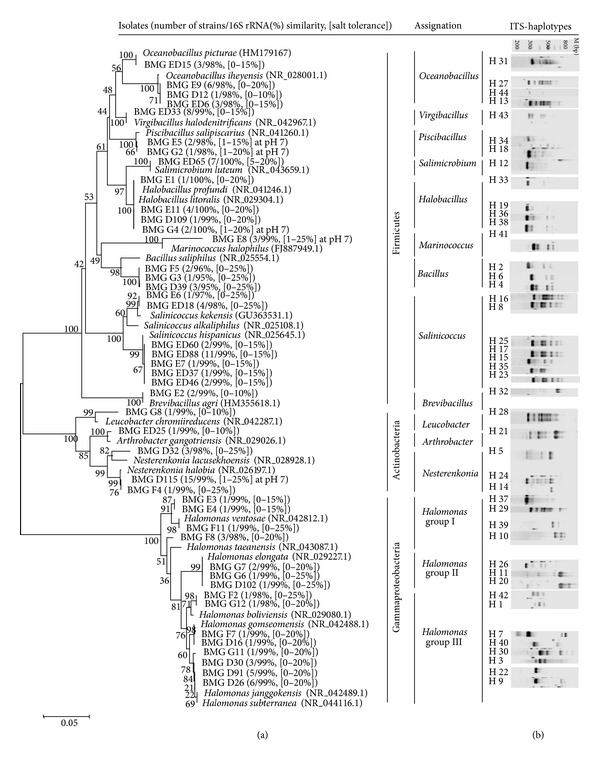
Phylogenetic diversity of haloalkaliphilic bacteria. (a) Unrooted phylogenetic tree of 44 partial 16S rRNA sequences (500 bp) of the arid saline system isolates with the 24 closest phylogenetic relatives. The method of Jukes and Cantor was used to calculate evolutionary distances and tree topology was constructed using MEGA 4.0. Bootstrap values (*n* = 1000 replicates) were indicated at the nodes. The number of isolates per ITS haplotype, the 16S rRNA similarity percentage (refseq rna database), and NaCl range for growth at pH 11 (or at pH 7 where mentioned), are indicated in parenthesis. (b) 16S–23S rRNA ITS haplotypes of 44 representative isolates as resolved on 2% agarose gels. ITS haplotype numbers are indicated. Lane M corresponds to a 100 bp ladder.

ITS fingerprinting method is a molecular tool based on the sequence and length heterogeneity of the bacterial rRNA operon 16S–23S intergenic spacer and provides a high phylogenetic resolution. It can discriminate bacterial isolates up to the subspecies level [26, 49, 50]. To manage the large set of isolates in our collection, ITS-PCR fingerprinting was applied as a first screening method. Among the 122 isolates, 44 distinct haplotypes (H1–H44) were detected. All profiles were composed by 1 up to 8 reproducible bands of approximate sizes ranging from 180 to 800 bp (Figure 2). 

The most encountered haplotype was ITS-H24 revealed in 15 strains isolated from sand and sediment samples collected from Ksar Ghilane, Chott el Djerid, and Sabkhet Ennaouel. Strains belonging to this haplotype were classified as strict halophile (at pH 7) and extremely halotolerant at alkaline pH (pH 10-11). The second most frequent represented pattern was haplotype ITS-H17 present in 11 strains isolated from *Salicornia* plants rhizosphere and algal biofilm collected from Sabkhet El Melah and Chott el Douz, respectively. These strains were found to be able to grow in media with 15% NaCl and pH ranging from 7 to 11. Other ITS haplotypes were frequently encountered like ITS-H43 in 8 isolates and ITS-H9, and H12 shown by 6 strains. The remaining ITS haplotypes were shown to be, in the major part, strains specific haplotypes (Table 1). 

Partial 16S rRNA gene sequencing was performed for representative isolates of each distinct haplotype (*n* = 44) and analyzed using BLAST. Phylogenetic analysis revealed that the isolates were allocated into thirteen different genera with an uneven distribution: *Halomonas, Salinicoccus, Nesterenkonia, Oceanobacillus, Virgibacillus, Halobacillus, Salimicrobium, Bacillus, Piscibacillus, Marinococcus, Brevibacillus, Leucobacter, *and *Arthrobacter*. They were placed into the three major bacteria phyla Firmicutes, Actinobacteria and Gammaproteobacteria. In a similar work, microbial diversity analysis in water and sediment of lake Chaka, a hypersaline lake on Tibetan plateau, permitted the assignation of bacterial community into the same three groups of Firmicutes, Gammaproteobacteria, and Actinobacteria [51].

The Firmicutes phyla including *Bacillus, Halobacillus, Piscibacillus, Oceanobacillus, Virgibacillus, Salimicrobium, Marinococcus *and *Salinicoccus *were more abundant and diverse. They constitute also the obligate haloalkaliphiles fraction at neutral pH (*Halobacillus*, *Piscibacillus*, and *Marinococcus*) and at all pH values (*Salimicrobium *that needs at least 5% NaCl to grow) (Table 2). Compared to similar studies carried out on salt lake [21, 44, 52], marine habitat [20, 53], and other hypersaline sediments [47] where limited number of genera were identified, arid saline systems of Tunisia revealed a highly diverse community. The isolates obtained from Alkaline Lonar lake in India were associated with the members of diverse *Bacillus* related genera (*Paenibacillus, Bacillus,* and *Alkalibacillus*) [7]. While in deep-sea hypersaline lakes, taxonomic analyses showed that two-thirds of 89 isolates were mostly representative of the genus *Bacillus* and the related genera *Halobacillus, Virgibacillus, *and* Pontibacillus *[54]. In comparison to these reports, *Bacillus *species are among the most commonly found aerobic, bacterial alkaliphiles, both in Soda lakes and in less selective environments [44, 55–58]. The same result was observed in other arid saline systems such as the Golea Salt lake in Algeria Sahara [52], Chott el Djerid [21, 44, 59] and Tunisian multipond solar saltern [18, 58, 59]. This high occurrence and the ability of *Bacillus* and *Bacillus* related genera to tolerate salt and alkaline stress prove that they are well adapted to arid-saline environments being physiologically active and not only present as dormant spores. Indeed, recent report indicated that they contribute to the system biological robustness and function [60]. Three Bacillaceae strains (BMG F5, BMG D39, and BMG G3) isolated from sediments and thermomineral water from Ksar Ghilane BDV1.8 site (a thermomineral natural pool) showed a very low 16S rRNA sequence homology (95-96%) with *Bacillus saliphilus* that was previously isolated from mineral pool in southern Italy [38]. The Ksar Ghilane strains could represent new alkaliphilic and extremely halotolerant species related to *B. saliphilus*, particularly adapted to high mineral concentrations in desert environment. 

Other species-microniche correlations are noteworthy. *Oceanobacillus iheyensis* strains (*n* = 10) were all isolated from salt crust samples, whereas *Halobacillus* (*n* = 8), *Piscibacillus* (*n* = 3), and *Salimicrobium* (*n* = 7) isolates were recovered from salty sediments and soils (Table 1). On the other hand, the 17 isolates identified as *Salinicoccus hispanicus*, 5 isolates of *Salinicoccus alkaliphilus,* and 3 isolates of *Marinococcus halophilus* were clearly associated with the rhizosphere of the desert plant *Salicornia* and algal biofilm. Whilst *Marinococcus halophilus* was recently described as a plant-growth promoting rhizospheric bacterium isolated from the same environment [21], this work constitutes the first report on the capabilities of haloalkaliphilic *Salinicoccus *species to colonize and thrive into the plant rhizosphere in desert environment.

The phylum Actinobacteria was represented by 4 species that belong to the Micrococcaceae family:* Nesterenkonia halobia* (16 isolates from Chott el Djerid, Sabkhet Ennaouel, and Ksar Ghilane; 99% of 16S rRNA sequence identity)*, Nesterenkonia lacusekhoensis* (3 isolates from Ksar Ghilane and Chott el Douz; 98% of identity)*, Leucobacter chromiireducens *(isolate BMG G8 from Sabkhet El Melah; 99% of identity), and* Arthrobacter gangotriensis* (isolate BMG ED25 from Sabkhet El Melah; 99% of identity). Species of the genus *Nesterenkonia *were previously reported as halotolerant and were isolated from different saline ecosystems like Brazilian Mangrove sediment [61] and hypersaline Ekho lake in East Antarctica [62]. *Nesterenkonia halobia* was also found as the unique Actinobacteria representative in *Salicornia* rhizosphere [21]. In the current prospection, *N. lacusekhoensis *and particularly* N. halobia* were recovered mainly from sand samples and showed changing halotolerance behavior at neutral and alkaline pH indicating a specific fine-tuned adaptation of these species to sand and salty sediments as ecological niche. With regard to the *Arthrobacter* species, they were previously reported as halotolerant and were isolated from east African soda lakes [63] and Antarctica [64]. Interestingly, the moderate halophile isolates *Leucobacter chromiireducens* and *Arthrobacter gangotriensis* are not known to be natural inhabitant of arid-saline systems.

Gram-negative bacteria were represented by a unique genus *Halomonas* counting 23.87% of the whole collection, in accordance with the recent work of Mapelli et al. [21]. *Halomonas* isolates were retrieved from all sample types, assigned to 7 distinct species and clustered within 3 phylogenetic groups: (i) *Halomonas* group I including *H. ventosae* (*n* = 3), and *H. taeanensis* (*n* = 3); (ii) *Halomonas* group II represented by *H. elongata* (*n* = 4); and (iii) *Halomonas* group III constituted by *H. boliviensis* (*n* = 2), *H. gomseomensis* (*n* = 2) and the related species *H. janggokensis* (*n* = 14) and *H. subterranea* (*n* = 1) (Table 1, Figure 2). Considering the nonmonophyletic status of the *Halomonas* genus and the need of a deep taxonomic revision [65], the number of recovered species indicates high intragenus diversity. In addition, there was no clear correlation between the recovered *Halomonas* species with their isolation origin, pointing out their adaptation capabilities to harsh conditions. Indeed, members of this genus have been isolated from diverse saline environments, including athalassohaline and thalassohaline Lakes and marine waters [20, 66]. However, by applying culture dependent and independent approaches [18, 20, 58], more diverse communities including bacteria from the *Alpha-, Beta-, Gamma-,* and *Deltaproteobacteria *subclasses were revealed in similar ecosystems like the Inner Mongolian Soda Lake [17] and the hyperalkaline spring waters in Jordan [67]. The limited number of Gram-negative bacteria detected in our hypersaline samples may be due to the enrichment and culturing procedure that favor the growth of Gram-positive bacteria, as reported earlier [7], and where fast-growing alkalitolerant *Halomonas sp*. outcompete other Gram negative microorganisms at different NaCl concentration and pH values [51]. 

### 3.3. Geographic Distribution and Microdiversity

**Figure 3:**
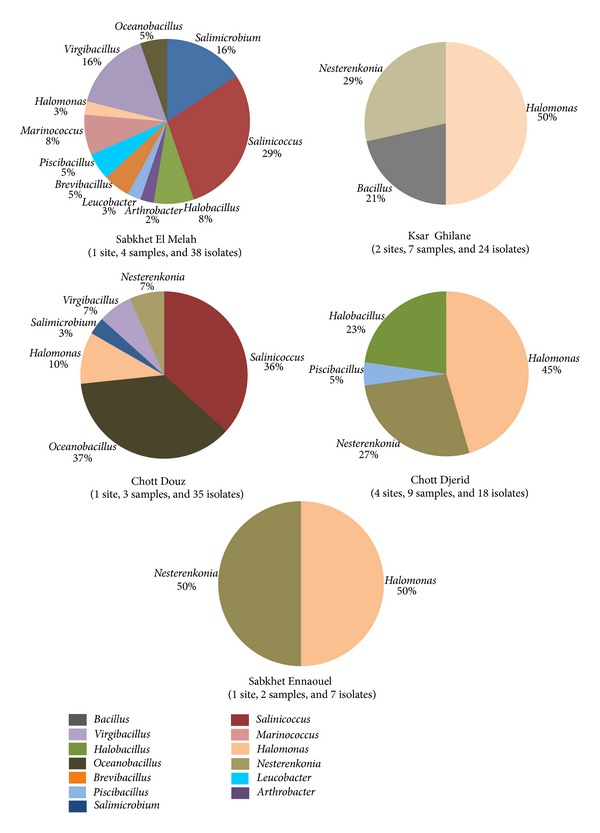
Geographic distribution of haloalkaliphilic bacteria isolated from natural saline systems of southern Tunisian Sahara.

Arid environment and saline systems in southern Tunisia are characterized by unstable climatic conditions, due to the periodic flooding by the subsurface ground water associated with high salt during dry phases. These specific conditions make such environment fascinating ecosystems to study the diversity and the ecological adaptations of thriving microorganisms. In the current study, cultivation approach showed a particular distribution of haloalkaliphilic bacteria according to their sampling origin (Table 1, Figure 3). The general distribution of the genera was very similar in Ksar Ghilane, Sabkhet Ennaouel, and Chott el Djerid with low bacterial diversity and the dominance of *Halomonas* and *Nesterenkonia* species (Figure 3). Beside the specific occurrence of *Nesterenkonia* species in these stations, *Bacillus saliphilus* and *Halobacillus profundi* were exclusively isolated from Ksar Ghilane and Chott el Djerid, respectively.

Sabkhet El Melah showed the most diverse community displaying a mixture of strains affiliated into 11 genera. Among them, *Arthrobacter*, *Leucobacter*, *Brevibacillus,* and *Marinococcus* were exclusively detected in this site. In contrast, we noted the absence of *Nesterenkonia* strains, frequently isolated from all the other sites (Figure 3). The high diversity detected in Sabkhet El Melah could be explained by its geographic location (a coastal saline system) that allows water exchange with the open sea. Indeed, the occurrence of *Marinococcus halophilus* (BMG E8 and two other isolates), a marine bacterium shown to be strict halophilic at neutral pH, may indicate that the observed diversity is of marine origin rather than terrestrial. Besides, *Leucobacter chromiireducens* was first isolated from activated sludge of a waste water treatment plant contaminated with chromium and was shown to be halotolerant and able to tolerate up to 5 mM Cr(VI) [68]. Likewise, *Arthrobacter gangotriensis* is closely related to *A. sulfurous* isolated from oil contaminated sludge and able to achieve desulphurization [69]. The presence in Sabkhet El Melah of *L. chromiireducens* and *A. gangotriensis* related species may indicate anthropogenic and industrial pollution due to their vicinity to an offshore oil field and oil harbor terminal.

Chott el Douz is most similar to Sabkhet El Melah in terms of diversity with 6 distinct detected genera: *Halomonas*, *Virgibacillus*, *Salimicrobium,* and *Nesterenkonia* and a marked dominance of bacteria assigned to *Oceanobacillus *(37%) and *Salinicoccus* (36%). Interestingly, all the isolates assigned to *Salinicoccus alkaliphilus* (*n* = 5, ITS haplotypes H8 and H16) and to *Oceanobacillus iheyensis* (*n* = 10, ITS haplotypes H13, H27, and H44) occurred specifically in this site (Table 1, Figure 3). In similar studies, *S. alkaliphilus* was isolated from salt lakes; however, *O. iheyensis* is a deep-sea bacterium with original genomic futures and adaptive capabilities to changing environments [5, 70, 71]. The high prevalence of *O. iheyensis* species in salt crust samples of Chott el Douz confirms its adaptation potential to such extreme ecosystem.

The adaptive capabilities of the dominating haloalkaliphile species detected in the current study could be, in part, inferred to their intraspecific microdiversity. This microdiversity is highlighted by the number of ITS haplotypes displayed by a single or a complex of bacterial species. *Salinicoccus hispanicus* isolates, shown to thrive in plant rhizosphere and algal biofilm, were clustered in 5 ITS haplotypes (H15, H17, H23, H25, and H35). As well, *Halomonas* isolates recovered from all the sites were allocated into seven different species and 15 ITS haplotypes. Within this genus, *Halomonas* group III includes the 3 closely related species with 6 distinct ITS haplotypes: *H. gomseomensis* (H7 and H40)*, H. janggokensis *(H2, H9, and H22) and* H. subterranea* (H30). Isolates of these species that could be considered as a single one [65, 72] were recovered from all the sites except from Sabkhet El Melah (Table 1, Figure 3). Their high level of microdiversity could contribute to their ecological fitness and their ability to adapt to desert and saline environments. Overall, the microdiversity is attributed to different combinations of DNA sequence blocks making the genome more competent to accumulate mutations, insertions, and deletions due to selective pressure. The exact contribution of the microdiversity to microbial adaptive strategies is not clearly elucidated. However, high extent of intraspecific polymorphism is usually shown by bacterial species that are well adapted and thriving in extreme environments [20, 26, 59]. 

### 3.4. Hydrolytic Activities of Isolates

Beside the bacterial diversity of the southern Tunisia ecosystem, the current study assesses the biotechnological potential of desert isolates. The occurrence of hydrolytic enzymes could be used as biochemical marker to judge the microbial heterogeneity among the selected haloalkaliphilic bacteria. The ability of producing four different hydrolytic enzymes was tested qualitatively for 44 identified strains in the optimum growth conditions (10% NaCl and pH 10). A total of 15, 17, 16, and 15 isolates were able to produce protease, lipase, DNase, and amylase, respectively (Table 2). It is interesting to note that combined hydrolytic activities were also detected in many strains. One strain, BMG D102, affiliated to *Halomonas elongata* showed all four enzyme activities (PGPR strain as Mapelli et al). Strains affiliated to *Bacillus saliphilus, Nesterenkonia halobia*, *Halobacillus litoralis*, *Piscibacillus salipiscarius,* and *Halobacillus profundi* were able to produce 3 hydrolytic activities. Sanchez-Porro and colleagues [73] showed the abundance of these hydrolytic enzymes produced by moderately halophilic bacteria. It is worth noting that Gram-positive bacteria showed more hydrolytic activities. Similar variations in the production of these enzymes were reported among the bacteria isolated from Howz Soltan lake in Iran and Pulicat Lake in India [15, 23]. 

Two hydrolytic activities were demonstrated by 13 isolates affiliated to *Halomonas, Halobacillus, Piscibacillus, Oceanobacillus, *and* Bacillus *genera. However, unique hydrolytic activity was detected in 12 strains assigned to *Halomonas, Salinicoccus, Piscibacillus, Virgibacillus, Oceanobacillus, *and* Marinococcus *genera. On the other hand, 11 isolates, members of *Nesterenkonia*, *Halomonas*, *Salinicoccus,* and *Arthrobacter* genera, did not show any activity. This absence may be due to the released hydrolase quantity, not sufficiently enough to cause visible clearing zone on the plates.

The majority of the enzyme producers were affiliated to the *Bacillus* and *Halomonas* genera. Lipase was produced by 38.6% of the isolates; DNase was shown by 36.3% of the strains. For protease and amylase, 34% of the selected strains were able to release these enzymes. Similar results were observed for species isolated from saline alkaline systems affiliated to *Halobacillus* sp. [74], *Nesterenkonia* sp. [75], *Virgibacillus* sp. [76], and *Bacillus* sp. [77]. The most active strains are able to produce at least 3 hydrolases, were isolated from Chott el Djerid, Ksar Ghilane, and Sabkhet El Melah, and were all extremely haloalkalitolerant bacteria. 

## 4. Conclusion

Our overall results indicate that haloalkaliphilic bacteria constitute an important part of the microbiota that inhabits arid and saline systems in southern Tunisia. A huge phenotypic and phylogenetic diversity was observed. Extremely haloalkalitolerant bacteria were the most dominant group and were affiliated to *Bacillus*, *Nesterenkonia*, *Salinicoccus,* and *Marinococcus* genera, of which several isolates could represent putative new species. A clear correlation between some species with specific ecological niches was also demonstrated. Besides, difference in the bacterial diversity rates between the studied sites was shown. The heterogeneity of haloalkaliphilic bacteria was confirmed by their hydrolytic enzymatic patterns variability including protease, lipase, DNase, and amylase. These enzymes are generally haloalkaliphilic which makes them interesting candidates to be employed in different industrial processes. The detected phenotypic and phylogenetic diversity points out that saline systems of southern Tunisia could represent a valuable source of new lineages and metabolites. 

## Figures and Tables

**Table 1:** Phylogenetic characterization of the haloalkaliphiles (ITS haplotype and 16S rRNA identification) and samples (code, nature, and location) collected from arid saline systems in southern Tunisia during February 2008 and 2010.

Representative strains of ITS haplotypes (number of isolates per haplotype)	Accession number	Phylogenetic group	Closet described species and identity (%)	Accession number	Sampling date	Sample code	Sampling sites (number of isolates per site)	Type of matrix
H1-BMG G12 (1)	KF179184	Proteobacteria	*Halomonas boliviensis* (98%)	EU_308325.1	February 2010	BDV1.8.A	Ksar Ghilane	Mud
H2-BMG F5 (2)	KF179185	Firmicutes	*Bacillus saliphilus *(96%)	HM_811185.1	February 2010	BDV1.8.B	Ksar Ghilane	Black sediment
H3-BMG D30 (3)	KF179190	Proteobacteria	*Halomonas janggokensis* (100%)	EU_308361.1	February 2010	BDV1.8.C	Ksar Ghilane	Thermomineral water
H4-BMG D39 (3)	KF179189	Firmicutes	*Bacillus saliphilus *(95%)	HM_811185.1	February 2010	BDV1.8.C	Ksar Ghilane	Thermomineral water
H5-BMG D32 (3)	KF179187	Actinobacteria	*Nesterenkonia lacusekhoensis* (98%)	GQ_064877.1	February 2010	BDV1.4 BDV6.2	Ksar Ghilane (1) Chottel Douz (2)	Sand with vegetation Salt crust
H6-BMG G3 (1)	KF179188	Firmicutes	*Bacillus saliphilus *(95%)	HM_811185.1	February 2010	BDV1.8.B	Ksar Ghilane	Black sediment
H7-BMG F7 (1)	KF179205	Proteobacteria	*Halomonas gomseomensis* (99%)	EU_308352.1	February 2010	BDV4.2	Sabkhet Ennaouel	Saline water
H8-BMG ED18 (4)	KF179213	Firmicutes	*Salinicoccus alkaliphilus* (98%)	GU_363531.1	February 2008	BDV6.3	Chottel Douz	Algal biofilm
H9-BMG D26 (6)	KF179191	Proteobacteria	*Halomonas janggokensis* (100%)	EU_308361.1	February 2010	BDV1.8.A BDV6.3	Ksar Ghilane (1) Chottel Douz (5)	Mud Algal biofilm
H10-BMG F8 (3)	KF179206	Proteobacteria	*Halomonas taeanensis *(98%)	HQ_190038	February 2010	BDV4.1 BDV17.3/18.3	Sabkhet Ennaouel (1) Chottel Djerid (2)	Sediment with salt crust Sediment with salt
H11-BMG G6 (1)	KF179174	Proteobacteria	*Halomonas elongata* (99%)	NR_029227.1	February 2010	BDV18.3	Chottel Djerid	Black sediment
H12-BMG ED65 (7)	KF179199	Firmicutes	*Salimicrobium luteum* (100%)	FJ_157154.1	February 2008	BDIII-11.A1/B1 BDV6.2	Sabkhet El Melah (6) Chottel Douz (1)	Sediment with salt Salt crust
H13-BMG ED6 (3)	KF179211	Firmicutes	*Oceanobacillus iheyensis* (98%)	GU_326361.1	February 2008	BDV6.2	Chottel Douz	Salt crust
H14-BMG F4 (1)	KF179175	Actinobacteria	*Nesterenkonia halobia* (99%)	EF_153433.1	February 2010	BDV20.1	Chottel Djerid	Sand
H15-BMG E7 (1)	KF179194	Firmicutes	*Salinicoccus hispanicus* (99%)	NR_025645.1	February 2008	BDIII-11.C3	Sabkhet El Melah	Salicornia rhizosphere
H16-BMG E6 (1)	KF179210	Firmicutes	*Salinicoccus alkaliphilus* (97%)	GU_363531.1	February 2008	BDV6.3	Chottel Douz	Algal biofilm
H17-BMG ED88 (11)	KF179193	Firmicutes	*Salinicoccus hispanicus* (99%)	NR_025645.1	February 2008	BDIII-11.C3 BDV6.3/BDV6.2	Sabkhet El Melah (7) Chottel Douz (4)	Salicornia rhizosphere Algal biofilm/salt crust
H18-BMG G2 (1)	KF179183	Firmicutes	*Piscibacillus salipiscarius* (98%)	HM_222702.1	February 2010	BDV19.6	Chottel Djerid	Sediment and salt
H19-BMG E11 (4)	KF179202	Firmicutes	*Halobacillus litoralis* (100%)	HM_636928.1	February 2008	BDIII-11.B1/A2 BDV20.2	Sabkhet El Melah (3) Chottel Djerid (1)	Sediment with salt crust Sediment (sandy soil)
H20-BMG D102 (1)	KF179176	Proteobacteria	*Halomonas elongata* (99%)	NR_029227.1	February 2010	BDV18.3	Chottel Djerid	Sediment
H21-BMG ED25 (1)	KF179200	Actinobacteria	*Arthrobacter gangotriensis* (99%)	FR_749771.1	February 2008	BDIII-11.A1	Sabkhet El Melah	Sediment with salt
H22-BMG D91 (5)	KF179178	Proteobacteria	*Halomonas janggokensis* (100%)	EU_308361.1	February 2010	BDV17.2 BDV1.8.A/1.8.C	Chottel Djerid (1) Ksar Ghilane (4)	Salt crust Mud/thermomineral water
H23-BMG ED46 (2)	KF179192	Firmicutes	*Salinicoccus hispanicus* (99%)	NR_025645.1	February 2008	BDIII-11.C3 BDV6.3	Sabkhet El Melah (1) Chottel Douz (1)	Salicornia rhizosphere Algal biofilm
H24-BMG D115 (15)	KF179177	Actinobacteria	*Nesterenkonia halobia* (99%)	EF_153433.1	February 2010	BDV20.1/19.6 BDV1.4 BDV4.2/4.1	Chottel Djerid (4) Ksar Ghilane (7) Sabkhet Ennaouel (4)	Sand Sand with vegetation Saline water/salt crust
H25-BMG ED60 (2)	KF179208	Firmicutes	*Salinicoccus hispanicus* (99%)	NR_025645.1	February 2008	BDV6.3 BDIII-11.C3	Chottel Douz (1) Sabkhet El Melah (1)	Algal biofilm Salicornia rhizosphere
H26-BMG G7 (2)	KF179182	Proteobacteria	*Halomonas elongata* (99%)	NR_029227.1	February 2010	BDV20.2	Chottel Djerid	Sediment
H27-BMG E9 (6)	KF179209	Firmicutes	*Oceanobacillus iheyensis* (98%)	HM_854234.1	February 2008	BDV6.2	Chottel Douz	Salt crust
H28-BMG G8 (1)	KF307740	Actinobacteria	*Leucobacter chromiireducens* (99%)	EF_153433.1	February 2008	BDIII-11.A2	Sabkhet El Melah	Sediment with water and salt crust
H29-BMG E4 (1)	KF179215	Proteobacteria	*Halomonas ventosae* (99%)	FM_210950.1	February 2008	BDV6.1	Chottel Douz	Saline water
H30-BMG G11 (1)	KF179180	Proteobacteria	*Halomonas subterranea* (99%)	EU_308353.1	February 2010	BDV17.3	Chottel Djerid	Sediment
H31-BMG ED15 (3)	KF179214	Firmicutes	*Oceanobacillus picturae* (98%)	HM_179167.1	February 2008	BDV6.3 BDIII-11.B1	Chottel Douz (2) Sabkhet El Melah (1)	Algal biofilm Sediment with salt
H32-BMG E2 (2)	KF179198	Firmicutes	*Brevibacillus agri* (99%)	HM_355618.1	February 2008	BDIII-11.A1	Sabkhet El Melah	Sediment with salt
H33-BMG E1 (1)	KF179203	Firmicutes	*Halobacillus litoralis* (100%)	HM_636928.1	February 2008	BDIII-11.A2	Sabkhet El Melah	Sediment with water and salt crust
H34-BMG E5 (2)	KF179197	Firmicutes	*Piscibacillus salipiscarius* (98%)	HM_222702.1	February 2008	BDIII-11.A1	Sabkhet El Melah	Sediment with salt
H35-BMG ED37 (1)	KF179195	Firmicutes	*Salinicoccus hispanicus* (99%)	NR_025645.1	February 2008	BDIII-11.C3	Sabkhet El Melah	Salicornia rhizosphere
H36-BMG D109 (1)	KF179179	Firmicutes	*Halobacillus profundi* (99%)	EU_482426.1	February 2010	BDV19.6	Chottel Djerid	Sediment and salt
H37-BMG E3 (1)	KF179207	Proteobacteria	*Halomonas ventosae* (99%)	FM_210950.1	February 2008	BDV6.1	Chottel Douz	Saline water
H38-BMG G4 (2)	KF179181	Firmicutes	*Halobacillus litoralis* (100%)	HM_636928.1	February 2010	BDV17.3	Chottel Djerid	Sediment
H39-BMG F11 (1)	KF179186	Proteobacteria	*Halomonas ventosae* (99%)	AB_617544.1	February 2010	BDV1.8.B	Ksar Ghilane	Black sediment
H40-BMG D16 (1)	KF179204	Proteobacteria	*Halomonas gomseomensis* (99%)	EU_308352.1	February 2010	BDV4.2	Sabkhet Ennaouel	Saline water
H41-BMG E8 (3)	KF307741	Firmicutes	*Marinococcus halophilus* (99%)	FJ_887949.1	February 2008	BDIII-11.C3	Sabkhet El Melah	Salicornia rhizosphere
H42-BMG F2 (1)	KF179201	Proteobacteria	*Halomonas boliviensis* (98%)	EU_308325.1	February 2008	BDIII-11.C3	Sabkhet El Melah	Salicornia rhizosphere
H43-BMG ED33 (8)	KF179196	Firmicutes	*Virgibacillus halodenitrificans* (99%)	AM_950296.1	February 2008	BDIII-11.B1/C3	Sabkhet El Melah (6)	Sediment with salt crust/salicornia rhizosphere
						BDV6.2	Chottel Douz (2)	Salt crust
H44-BMG D12 (1)	KF179212	Firmicutes	*Oceanobacillus iheyensis* (98%)	HM_854234.1	February 2008	BDV6.2	Chottel Douz	Salt crust

**Table 2:** Salt and pH tolerance levels and hydrolytic activities of ITS haplotype representatives isolates.

Representative strains of ITS haplotypes	Identification	Gram	NaCl tolerance range (%)	pH tolerance range	Production of extracellular enzymes			
					Protease	Lipase	DNase	Amylase
H1-BMG G12	*Halomonas boliviensis *	−	0–20 ± 0.1	7–11 ± 0.2	−	+	+	−
H2-BMG F5	*Bacillus saliphilus *	+	0–25 ± 0.1	7–11 ± 0.2	−	−	+	+
H3-BMG D30	*Halomanas janggokensis *	−	0–20 ± 0.1	7–11 ± 0.2	−	+	−	−
H4-BMG D39	*Bacillus saliphilus *	+	0–25 ± 0.1	7–11 ± 0.2	+	−	+	+
H5-BMG D32	*Nesterenkonia lacusekhoensis *	+	0–25 ± 0.1	7–11 ± 0.2	−	−	−	−
H6-BMG G3	*Bacillus saliphilus *	+	0–25 ± 0.1	7–11 ± 0.2	+	−	+	+
H7-BMG F7	*Halomonas gomseomensis *	−	0–20 ± 0.1	7–11 ± 0.2	−	+	+	−
H8-BMG ED18	*Salinicoccus alkaliphilus *	+	0–25 ± 0.1	7–11 ± 0.2	+	–	−	−
H9-BMG D26	*Halomanas janggokensis *	−	0–20 ± 0.1	7–11 ± 0.2	−	+	−	−
H10-BMG F8	*Halomonas taeanensis *	−	0–20 ± 0.1	7–11 ± 0.2	−	–	−	−
H11-BMG G6	*Halomonas elongata *	−	0–25 ± 0.1	7–11 ± 0.2	−	+	−	+
H12-BMG ED65	*Salimicrobium luteum *	+	5–20 ± 0.1	7–11 ± 0.2	−	+	−	−
H13-BMG ED6	*Oceanobacillus iheyensis *	+	0–15 ± 0.1	7–11 ± 0.2	+	–	+	–
H14-BMG F4	*Nesterenkonia halobia *	+	0–25 ± 0.1	7–11 ± 0.2	+	+	−	+
H15-BMG E7	*Salinicoccus hispanicus *	+	0–15 ± 0.1	7–11 ± 0.2	−	−	−	−
H16-BMG E6	*Salinicoccus alkaliphilus *	+	0–25 ± 0.1	7–11 ± 0.2	−	−	−	−
H17-BMG ED88	*Salinicoccus hispanicus *	+	0–15 ± 0.1	7–11 ± 0.2	−	−	−	−
H18-BMG G2	*Piscibacillus salipiscarius *	+	1–20 ± 0.1 at pH 7 0–20 ± 0.1 at pH 10-11	7–11 ± 0.2	−	+	−	+
H19-BMG E11	*Halobacillus litoralis *	+	0–20 ± 0.1	7–11 ± 0.2	+	−	+	+
H20-BMG D102	*Halomonas elongata *	−	0–25 ± 0.1	7–11 ± 0.2	+	+	+	+
H21-BMG ED25	*Arthrobacter gangotriensis *	+	0–10 ± 0.1	7–11 ± 0.2	−	−	−	−
H22-BMG D91	*Halomanas janggokensis *	−	0–20 ± 0.1	7–11 ± 0.2	−	+	−	−
H23-BMG ED46	*Salinicoccus hispanicus *	+	0–15 ± 0.1	7–11 ± 0.2	−	−	−	−
H24-BMG D115	*Nesterenkonia halobia *	+	1–25 ± 0.1 at pH 7 0–25 ± 0.1 at pH 10-11	7–11 ± 0.2	−	+	−	+
H25-BMG ED60	*Salinicoccus hispanicus *	+	0–15 ± 0.1	7–11 ± 0.2	+	−	−	−
H26-BMG G7	*Halomonas elongata *	−	0–20 ± 0.1	7–11 ± 0.2	−	+	−	+
H27-BMG E9	*Oceanobacillus iheyensis *	+	0–20 ± 0.1	7–11 ± 0.2	+	−	+	−
H28-BMG G8	*Leucobacter chromiireducens *	+	0–10 ± 0.1	7–11 ± 0.2	−	+	−	+
H29-BMG E4	*Halomonas ventosae *	−	0–15 ± 0.1	7–11 ± 0.2	+	−	−	−
H30-BMG G11	*Halomonas subterranea *	−	0–20 ± 0.1	7–11 ± 0.2	−	+	−	−
H31-BMG ED15	*Oceanobacillus picturae *	+	0–15 ± 0.1	7–11 ± 0.2	+	−	−	−
H32-BMG E2	*Brevibacillus agri *	+	0–10 ± 0.1	7–11 ± 0.2	−	−	−	−
H33-BMG E1	*Halobacillus litoralis *	+	0–20 ± 0.1	7–11 ± 0.2	+	−	+	+
H34-BMG E5	*Piscibacillus salipiscarius *	+	1–15 ± 0.1 at pH 7 0–15 ± 0.1 at pH 10-11	7–11 ± 0.2	+	+	−	+
H35-BMG ED37	*Salinicoccus hispanicus *	+	0–15 ± 0.1	7–11 ± 0.2	−	−	−	−
H36-BMG D109	*Halobacillus profundi *	+	0–20 ± 0.1	7–11 ± 0.2	+	−	+	+
H37-BMG E3	*Halomonas ventosae *	−	0–15 ± 0.1	7–11 ± 0.2	−	−	−	−
H38-BMG G4	*Halobacillus litoralis *	+	1–20 ± 0.1 at pH 7 0–20 ± 0.1 at pH 10-11	7–11 ± 0.2	−	−	+	+
H39-BMG F11	*Halomonas ventosae *	−	0–25 ± 0.1	7–11 ± 0.2	−	−	−	−
H40-BMG D16	*Halomonas gomseomensis *	−	0–20 ± 0.1	7–11 ± 0.2	−	+	+	−
H41-BMG E8	*Marinococcus halophilus *	+	1–25 ± 0.1 at pH 7 0–25 ± 0.1 at pH 10-11	7–11 ± 0.2	−	−	+	−
H42-BMG F2	*Halomonas boliviensis *	−	0–25 ± 0.1	7–11 ± 0.2	−	+	+	−
H43-BMG ED33	*Virgibacillus halodenitrificans *	+	0–15 ± 0.1	7–11 ± 0.2	+	−	−	−
H44-BMG D12	*Oceanobacillus iheyensis *	+	0–10 ± 0.1	7–11 ± 0.2	−	−	+	−
